# Countrywide introduction of pulsed field ablation for the treatment of atrial fibrillation: Acute results from the FRANCE-PFA registry

**DOI:** 10.1016/j.hroo.2025.04.005

**Published:** 2025-04-25

**Authors:** Corentin Chaumont, Mikael Laredo, Olivier Thomas, Philippe Maury, Grégoire Massoulié, Pascal Defaye, Serge Boveda, Eloi Marijon, William Escande, Antoine Roux, Franck Raczka, Yves Guyomar, Pierre Jaïs, Pierre Ollitrault, Mathieu Granier, Samir Fareh, Hugo Marchand, Bertrand Pierre, Selim Abbey, Antoine Noel, Antoine Da Costa, Fabrice Extramiana, Olivier Cesari, Laure Champ-Rigot, Ziad Khoueiry, Thibault Villemin, Sarah Traullé, Frédéric Treguer, Hugues Bader, Jean-Claude Deharo, Bruno Degand, Benoît Guy-Moyat, Didier Klug, Estelle Gandjbakhch, Alexandre Zhao, Maxime Beneyto, Romain Eschalier, Sandrine Venier, Stéphane Combes, Antoine Lepillier, Marc Mielczarek, Nicolas Clementy, Aymeric Menet, Frédéric Sacher, Christian De Chillou, Frédéric Anselme

**Affiliations:** 1 Department of Cardiology, Rouen University Hospital and UNIROUEN, INSERM U1096, Rouen, France; 2 Institut de Cardiologie, Hôpital Pitié-Salpêtrière, AP-HP Sorbonne Université, Paris, France; 3 GHP Ambroise Paré Hartmann, Neuilly sur Seine, France; 4 Department of Cardiology, Toulouse University Hospital, Toulouse, France; 5 Cardiology Department, CHU Clermont-Ferrand, Clermont-Ferrand, France; 6 Cardiology Department, Grenoble-Alpes University, University Hospital, Grenoble, France; 7 Heart Rhythm Management Department, Clinique Pasteur, Toulouse, France; 8 European Georges Pompidou Hospital, Paris, France; 9 Centre Cardiologique du Nord, Saint-Denis, France; 10 Pôle Santé République, Clermont-Ferrand, France; 11 Clinique du Millénaire, Montpellier, France; 12 GHICL Hôpital Saint Philibert, Lomme, France; 13 IHU Liryc, Univ. Bordeaux, INSERM 1045, CHU de Bordeaux, Cardiac Arrhythmia Department, Bordeaux, France; 14 Department of Cardiology, Caen University Hospital, Caen, France; 15 CHU de Montpellier, Montpellier, France; 16 Hôpital de la Croix Rousse, Lyon, France; 17 Institut Arnault Tzanck, Saint-Laurent Du Var, France; 18 CHU de Tours, Tours, France; 19 Hôpital Privé du Confluent, Nantes, France; 20 Polyclinique Saint-Laurent, Rennes, France; 21 CHU de Saint-Etienne, Saint-Etienne, France; 22 Department of Cardiology, AP-HP Hôpital Bichat, Paris, France; 23 Clinique Saint Augustin, Bordeaux, France; 24 Clinique Saint-Pierre, Perpignan, France; 25 Department of Cardiology, Polyclinique Reims-Bezannes, Bezannes, France; 26 Clinique Victor Pauchet, Amiens, France; 27 Clinique Saint-Joseph, Trelaze, France; 28 Centre Hospitalier de Pau, Pau, France; 29 CHU de Marseille, Marseille, France; 30 CHU de Poitiers, Poitiers, France; 31 CHU de Limoges, Limoges, France; 32 CHU de Lille, Lille, France; 33 Department of Cardiology, University Hospital Nancy, Nancy, France

**Keywords:** Atrial fibrillation, Catheter ablation, Pulsed field ablation, Pulmonary vein isolation, Registry

## Abstract

**Background:**

Most data on atrial fibrillation (AF) ablation using the first available pentaspline pulsed field ablation (PFA) catheter (Farapulse, Boston Scientific Inc) come from retrospective center-level registries collected in highly experienced centers.

**Objective:**

This study aimed to provide exhaustive and prospective patient-level data on this new ablation modality.

**Methods:**

FRANCE-PFA is a nationwide registry (NCT06497933) that included all patients undergoing a first AF ablation using the pentaspline PFA catheter since the introduction of this technology in France. All French centers using this technology participated. Procedural data were prospectively collected at a patient level.

**Results:**

This registry included 5223 patients from 33 centers between March 2021 and February 2024 (mean age 65 ± 11 years, 55.4% paroxysmal AF). The procedure duration was 54 ± 23 minutes. Acute pulmonary vein isolation was achieved in 5211 patients (99.8%). The total number of PFA applications was 50 ± 22 with >70 applications in 746 patients (14.3%). Pulmonary vein isolation only was performed in 64.7% of patients (82.7% of paroxysmal AF, 44.5% of persistent AF, and 26.6% of long-standing persistent AF). The most common location for additional PFA lesion sets was the left atrium posterior wall in 1335 patients (25.6%), left atrium roof in 999 patients (19.1%), and mitral isthmus in 514 patients (9.8%). Major complications occurred in 50 patients (0.96%), with no esophageal complication or symptomatic phrenic nerve palsy reported in past hospital discharge.

**Conclusion:**

In this prospective and nationwide registry, AF ablation using the pentaspline PFA catheter seemed to be safe and acutely efficient, despite considerable heterogeneity in the number of patients treated at each center.

**Trial Registration:**

NCT06497933.

## Graphical abstract

**Figure:**
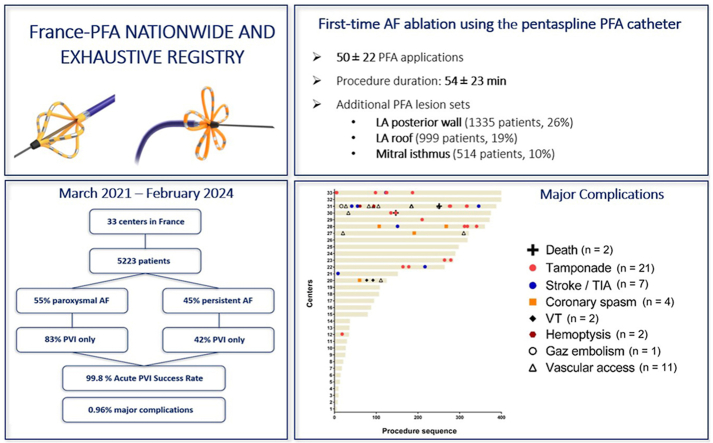

**Key Findings**

▪ FRANCE PFA is the first exhaustive, prospective, nationwide registry on atrial fibrillation (AF) ablation using the pentaspline pulsed field ablation (PFA) catheter.
▪ Our findings confirm the high acute efficacy and safety of PFA, even when used across centers with varying levels of experience.
▪ The data offer valuable insights into real-world clinical practices. Notably, 70% of the patients received more PFA applications per vein than initially recommended. The versatility and safety profile of PFA have also led to its use beyond pulmonary vein isolation, particularly in cases of persistent AF (58%) and, to a lesser extent, paroxysmal AF (17% of the cases).

## Introduction

Clinical guidelines for atrial fibrillation (AF) recommend pulmonary vein isolation (PVI) as the cornerstone of AF catheter ablation.^1^^,^^2^ Given the increase in the prevalence of AF, there is a growing need for safer and more efficient PVI procedures.^2^^,^^3^ Recently, pulsed field ablation (PFA) has emerged as a novel, nonthermal energy with high selectivity for atrial myocardial tissue.^4^ Recent data suggested that PFA has an excellent safety profile^5^^,^^6^ and may improve on previous “single-shot” ablation modalities.^7^ Most available data on AF ablation with the pentaspline PFA catheter (Farapulse, Boston Scientific Inc, Marlborough, MA) come from retrospective center-level registries at highly experienced centers.^5^^,^^6^ These results may not be applicable to a nationwide context or to broader use of PFA in routine clinical practice. Since the initial registries, practices regarding lesion sets and the number of PFA applications have evolved as the technique has expanded and centers have gained more experience.^8^, ^9^, ^10^ These changes have also revealed unexpected complications.^11^^,^^12^ The FRANCE-PFA registry aims to provide nationwide, exhaustive, and prospective patient-level data on this new catheter ablation modality. Here, we report on the ablation modalities and the acute safety and efficacy data from this registry.

## Methods

### Registry design

FRANCE-PFA was a prospective, nationwide registry (NCT06497933) that included all patients undergoing a first-time AF ablation using the pentaspline PFA catheter since the start of use of this technology in France in March 2021. Patients undergoing redo AF ablation procedures were not included. All French centers, academic or nonacademic, public or private, using this technology participated in the registry.

### Procedural details

Procedures were performed with uninterrupted oral anticoagulation. The left atrial appendage was assessed for potential thrombus preprocedurally by computed tomography (CT) scan or transesophageal echocardiogram. All procedures were conducted under general anesthesia with endotracheal intubation or laryngeal mask or under deep sedation (spontaneous ventilation). The Farapulse PFA system delivered high-voltage pulses at 2 kV per application, each lasting 2.5 seconds. Two catheter sizes (31 and 35 mm) were available for PVI. After the patients received heparin and achieved an activated clotting time ≥300 seconds, a 12-Fr multi-electrode pentaspline PFA catheter was advanced into the left atrium (LA) through a 13-Fr deflectable sheath (Faradrive, Boston Scientific Inc). The catheter was positioned over a guidewire (either straight or J shaped) to achieve circumferential contact at the PV antra. The initially recommended PVI protocol consisted of pairs of energy applications: 2 applications were performed in the “basket” configuration, followed by a slight rotation of the catheter (30°–40°) before delivering 2 additional applications. This ablation sequence was repeated in the “flower” configuration and applied at each PV (32 applications in total). Additional PFA applications at the PV antra, LA roof, posterior LA wall, mitral isthmus (MI), superior vena cava (SVC), and/or cavotricuspid isthmus (CTI) were left to the discretion of the operator. PVI was confirmed by the absence of discrete PV or atrial potentials at the PV antra, using the Farawave catheter. PV stimulation to test for exit block was not systematically performed. In rare cases, a 3-dimensional mapping system was used during the procedure. Procedure time was defined as the duration from vascular access to catheter removal, and dwell time referred to the time with the catheter(s) in the LA.

### Data collection

Procedural details, acute efficacy, and adverse events were prospectively collected in an online database (ORECA/France-PFA). Each center was asked to confirm and provide details on the occurrence of complications, which were then reviewed and adjudicated by an independent committee.

### Questionnaire

At the end of the inclusion period, a questionnaire was distributed to participating centers to assess key aspects of PFA implementation across different sites.

### Statistical analysis

Statistical analysis was mainly descriptive. Continuous variables were expressed as mean ± standard deviation for normally distributed data or median and interquartile range for non-normal data. Categorical variables were expressed as counts and percentage. Comparisons were performed by the Student *t* test for continuous variables and χ^2^ test for categorical variables. All tests were 2-sided, with a *P* < .05 considered statistically significant. Analyses were performed using SPSS software (version 29.0; IBM Corp, Armonk, NY).

## Results

### Enrollment

**Figure 1:**
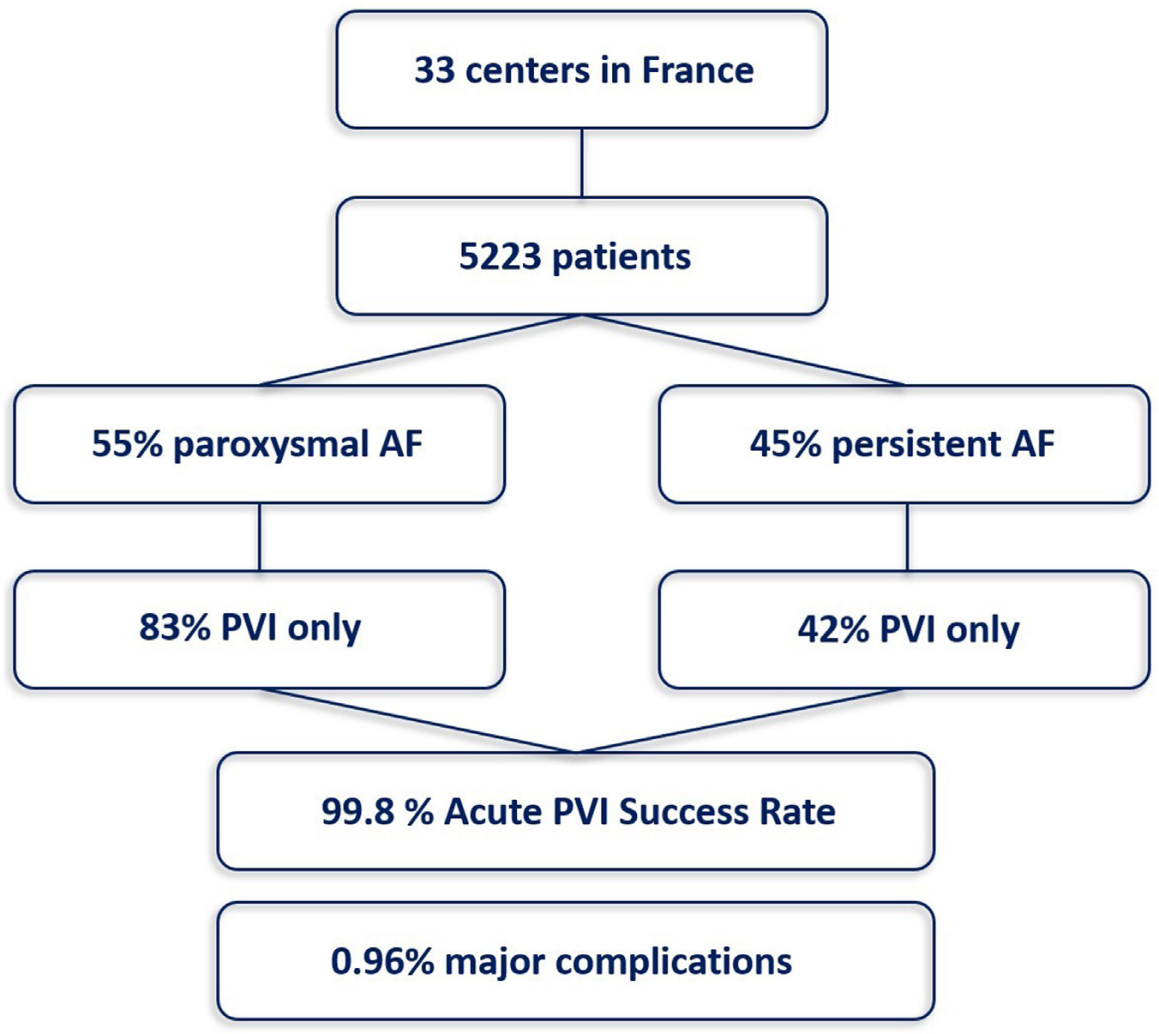
Flowchart. AF = atrial fibrillation; PVI = pulmonary vein isolation.

**Figure 2:**
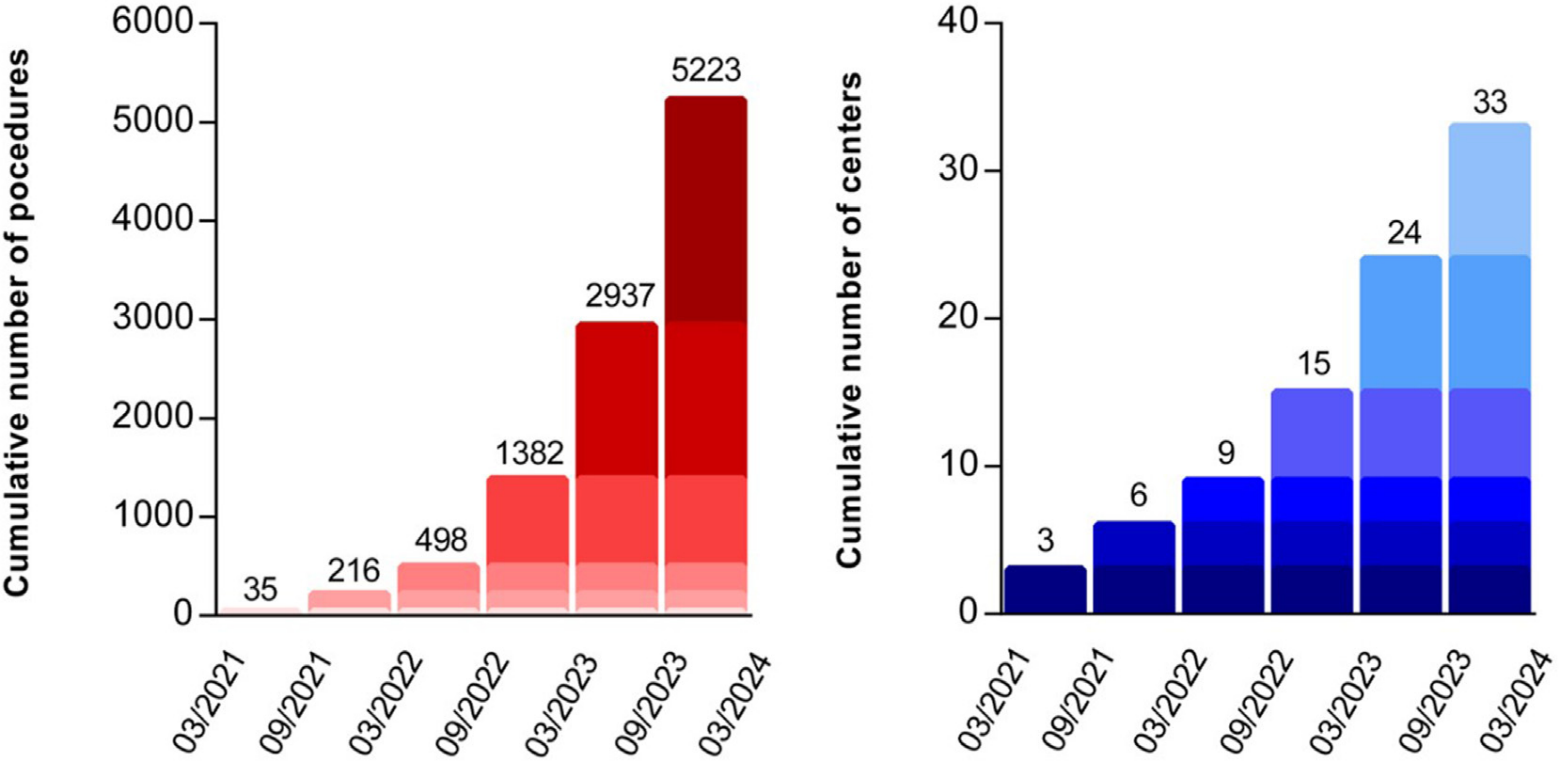
Trend in inclusions and participating centers over time. Note the strong progression in the use of PFA in France, with most procedures (80%) performed in 2023 and early 2024. More than half of the centers (18 of 33) have adopted PFA after March 2023. PFA = pulsed field ablation.

This registry included 5223 patients in 33 centers between March 2021 and February 2024 (Figure 1). Patients’ baseline demographics are indicated in Table 1. The mean age was 65.0 ± 11.2 years, and 30.6% of the patients were female. The indication for AF catheter ablation was paroxysmal AF in 55.4% of patients, persistent AF in 38.1%, and long-standing (LS) persistent AF in 6.4%. The trend in inclusions over time showed a strong progression in the use of PFA in France, with most procedures (80%) performed in 2023 and early 2024. More than half of the centers (18/33) have adopted PFA after March 2023 (Figure 2). The average number of patients treated by center was 158 ± 154 (range 6–456).

**Table 1:** Baseline patient characteristics

Parameters	Patients with available data	Value, mean ± SD or n (%)
Age (y)	5223 (100%)	65.0 ± 11.2
Female sex	5223 (100%)	1599 (30.6)
BMI (kg/m^2^)	5122 (98.1%)	27.7 ± 5.1
CHA_2_DS_2_-VASC score	5223 (100%)	1.9 ± 1.4
Hypertension	5223 (100%)	2004 (38.3)
Diabetes	5223 (100%)	551 (10.5)
History of stroke/TIA	5223 (100%)	302 (5.8)
Heart failure	5223 (100%)	689 (13.2)
Coronary artery disease	5223 (100%)	598 (11.4)
Type of AF		
Paroxysmal AF	4983 (95.4%)	2763 (55.4)
Persistent AF	4983 (95.4%)	1900 (38.1)
LS-persistent AF	4983 (95.4%)	320 (6.4)
Echocardiography parameters LVEF		
LVEF >50%	3926 (75.2%)	3053 (77.8)
LVEF 40%–50%	3926 (75.2%)	450 (11.5)
LVEF <40%	3926 (75.2%)	423 (10.8)
Echocardiography parameters		
LA size∗		
Normal	3204 (61.3%)	1380 (43.1)
Moderate enlargement	3204 (61.3%)	1308 (40.8)
Severe enlargement	3204 (61.3%)	516 (16.1)
Medications		
Class I	5223 (100%)	1110 (21.3)
Amiodarone	5223 (100%)	1744 (33.4)
Sotalol	5223 (100%)	280 (5.4)
Vitamin K antagonist	5223 (100%)	497 (9.5)
DOAC	5223 (100%)	4710 (90.2)

AF = atrial fibrillation; BMI = body mass index; CHA_2_DS_2_-VASC = congestive heart failure, hypertension, age ≥75 years, diabetes mellitus, prior stroke or transient ischemic attack or thromboembolism, vascular disease, age 65–74 years, sex category; DOAC = direct oral anticoagulant; LA = left atrium; LVEF = left ventricular ejection fraction; SD = standard deviation; TIA = transient ischemic attack.

∗ Normal LA size: diameter <42 mm and volume <34 mL/m^2^; moderate enlargement: diameter 42–50 mm and/or volume 34–40 mL/m^2^; severe enlargement: diameter >50 mm and/or volume >40 mL/m^2^.

### Procedural data

Most procedures were performed under general anesthesia (94.4%) (Table 2). All procedures were performed with a fluoroscopy-guided approach. A 3-dimensional mapping system was also used in 2.5% of the patients. The use of 31-mm Farawave catheters was largely predominant (86.5%). The mean procedure duration was 54 ± 23 minutes, dwell time was 34 ± 17 minutes, and fluoroscopy time was 16 ± 12 minutes. Procedure and fluoroscopy times were significantly longer in patients with additional lesions beyond PVI (60 ± 25 minutes vs 51 ± 21 minutes [*P* < .001] and 18 ± 16 minutes vs 15 ± 8 minutes [*P* < .001]). Considering PVI-only procedures and broken-down consecutive procedures by center into tertiles (51 first procedures/center, 52–148 procedures/center, and ≥149 procedures/center), we observed a modest reduction in procedure (53 ± 23 minutes, 52 ± 21 minutes, and 48 ± 18 minutes, for the first, second, and third tertiles, respectively) and fluoroscopy times (16 ± 9 minutes, 15 ± 9 minutes, and 13 ± 7 minutes, respectively).

**Table 2:** Procedural characteristics

Parameters	Patients with available data n (%) or n/N (%)	Value mean ± SD or (%)
Sedation technique		
General anesthesia	5223 (100%)	4931 (94.4%)
Deep sedation	5223 (100%)	292 (5.6%)
Use of 3D mapping system	5223 (100%)	133 (2.5%)
PVI success rate	5223 (100%)	5211 (99.8%)
Ablation device used		
35 mm	5123 (98.1%)	680 (13.3%)
31 mm	5123 (98.1%)	4443 (86.7%)
Skin-to-skin procedure time (min)	4983 (95.4%)	54 ± 23
Dwell time (min)	4223 (80.9%)	34 ± 17
Fluoroscopy time (min)	4942 (94.6%)	16 ± 12
Procedure time—PVI only (min)	3214 (95.1%)∗	51 ± 21
Fluoroscopy time—PVI only (min)	3199 (94.6%)∗	15 ± 8
Procedure time—PVI + (min)	1769 (96.0%)†	60 ± 25
Fluoroscopy time—PVI + (min)	1743 (94.6%)†	18 ± 16
PVI-only ablation (overall)	5223 (100%)	3380 (64.7%)
PVI only ablation (paroxysmal AF patients)	2763 (100%)	2285 (82.7%)
PVI only ablation (persistent AF patients)	1900 (100%)	845 (44.5%)
PVI only ablation (LS-persistent AF patients)	320 (100%)	85 (26.6%)
PVI only ablation (unknown type of AF)	240 (100%)	165 (68.8%)
Additional lesion sets		
Roof line	5223 (100%)	999 (19.1%)
No. of PFA applications	903/999 (90.4%)	11 ± 6
Mitral isthmus line	5223 (100%)	514 (9.8%)
No. of PFA applications	476/514 (92.6%)	17 ± 12
Left atrial posterior wall	5223 (100%)	1335 (25.6%)
No. of PFA applications	1263/1335 (94.6%)	16 ± 8
Superior vena cava	5223 (100%)	144 (2.7%)
No. of PFA applications	81/144 (56.3%)	5 ± 1
Cavotricuspid isthmus	5223 (100%)	145 (2.8%)

3D = 3-dimensional; AF = atrial fibrillation; LS = long-standing; PFA = pulsed field ablation; PVI = pulmonary vein isolation; SD = standard deviation.

∗ Among patients with PVI only procedures (n = 3380).

† Among patients with additional PFA lesions beyond PVI (PVI +) (n = 1843).

### Lesion sets and acute effectiveness

Acute PVI was achieved in 5211 patients (99.8%). In 8 patients, the ablation procedure was stopped before complete PVI because of hemodynamic impairment (tamponade in 6, vascular complication in 1, and stroke in 1). In the remaining 4 patients, right inferior PV could not be isolated due to inability to correctly position the PFA catheter.

PVI only was performed in 3380 patients (64.7%–82.7% of patients with paroxysmal AF, 44.5% of patients with persistent AF, and 26.6% of patients with LS-persistent AF). The number of applications for PVI was 42 ± 12, and 70% of patients received more PV applications than the standard PVI workflow (ie, 8 applications per vein—32 applications in total).

The overall number of PFA applications per patient was 50 ± 22 with >70 applications in 746 patients (14.3%). The most common location for additional PFA lesion sets was LA posterior wall in 1335 patients (26%—mean of 16 ± 8 PFA applications), LA roof in 999 patients (19%—mean of 11 ± 6 applications), and MI in 514 patients (10%—mean of 17 ± 12 applications). Additional PFA applications were also delivered along the CTI in 145 patients (2.8%). SVC isolation was performed using PFA in 144 patients (2.7%—mean of 5 ± 1 applications).

### Safety

**Figure 3:**
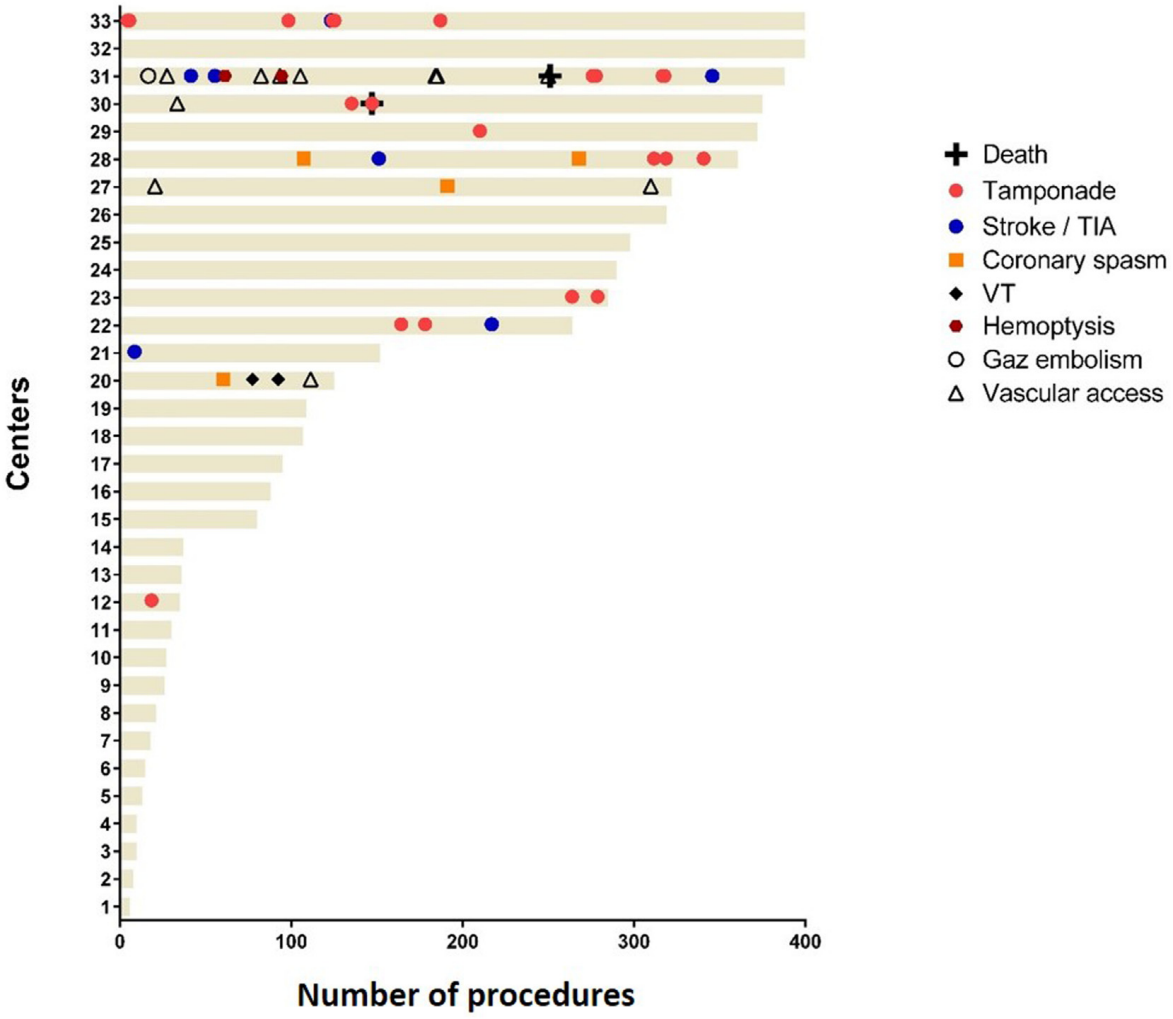
Occurrence of major complications over time at each center. The horizontal lines represent the number of patients treated at each of the 33 centers. Complications are displayed per center in chronological order. TIA = transient ischemic attack; VT = ventricular tachycardia.

Major complications occurred in 50 patients (0.96%) (Figure 3). Two patients died (0.04%), one from tamponade and another patient on the day after the procedure from refractory heart failure. The latter patient was admitted for terminal heart failure before the procedure. Other major complications included tamponade in 21 patients (0.4%), requiring percutaneous drainage in 13 (0.2%) and surgical intervention in 8 (0.2%). Stroke occurred in 5 patients (0.1%–2 with sequelae) with thrombolysis therapy administered to 1. Transient ischemic attack (TIA) was diagnosed in 2 patients (0.04%). All 7 patients with strokes or TIAs were compliant with their anticoagulant therapy, and no thrombus was found in preprocedure CT scans or transesophageal echocardiogram. The Farawave catheter was not withdrawn and reinserted into the LA in any of these patients during the procedure.

In 1 patient, a widening of the QRS followed by cardiac asystole was observed, with recovery within a minute. This episode was thought to be due to coronary gas embolism. Coronary spasm with ST-segment elevation occurred in 4 patients (0.08% of total cohort—0.6% of 659 patients who underwent mitral/CTI ablation) after PFA was applied in a “flower” configuration along the MI. Coronary angiography was performed on 2 of these patients, which showed coronary narrowing resolving after intracoronary nitroglycerin injection (1 mg). In the other 2 patients, the ST-segment elevation resolved completely within 1 minute after intravenous injection of a 3-mg bolus of nitroglycerin.

Ventricular tachycardia occurred in 2 patients (0.04%) with stable coronary artery disease, one during catheter manipulation in the LA and the other during PFA application on the posterior wall in a “flower configuration.” Vascular complications requiring intervention occurred in 11 patients (0.2%—surgical intervention in 4 patients and endovascular treatment in 7). Hemoptysis with arterial blood in the ventilation system was observed in 2 procedures (0.04%), notably performed using a straight guidewire. The hemoptysis resolved after heparin was reversed with protamine. Thoracic CT scans performed after the procedures revealed intrapulmonary hemorrhage in the left superior pulmonary vein in both patients. Both patients were asymptomatic and discharged with oral anticoagulation 1- and 3 days after the procedure. Follow-up thoracic CT scans at 1 month showed complete resolution of the initial hemorrhage. In a patient with dual-chamber pacemaker, a transient atrial loss of capture was observed after PFA application in the vicinity of the atrial lead. The pacing threshold returned to baseline within 24 hours.

When the major complications were analyzed over time (Figure 3), no trend toward fewer complications with increasing experience was observed (18/2155 during the first 100 procedure per center vs 32/3068 after the 100th procedure/center; *P* = .4).

Minor complications included transient complete AV block in 2 patients (0.04%), both resolving within 1 minute. The first was due to RBB injury during catheter manipulation in a patient with baseline LBBB. The second patient developed complete AV block during PFA application on the LSPV, likely of vagal origin, with spontaneous recovery.

No esophageal complications or symptomatic phrenic nerve (PN) palsy were reported beyond hospital discharge. Transient PN palsy was observed in 2 patients (0.04%) after PFA application on the RSPV in a “flower” configuration. Diagnosis was based on radiograph images in 1 patient with spontaneous ventilation (deep sedation), and recovery of normal diaphragmatic contraction was observed 3 minutes after the last application. For the second patient, diagnosis was based on PN pacing. The latter exhibited incomplete recovery of diaphragmatic contraction at the end of the procedure, with normal inspiration or expiration on radiograph before discharge and no other symptoms.

### Questionnaire

**Figure 4:**
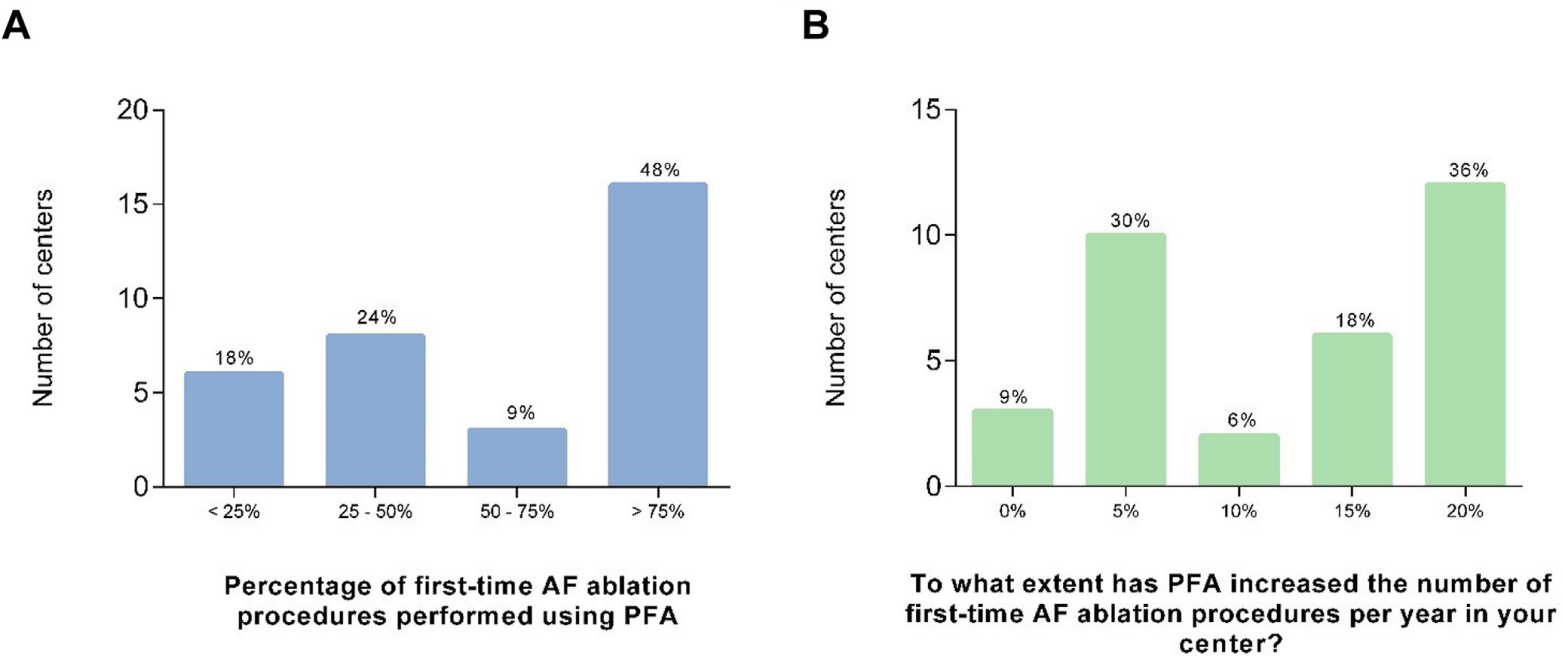
Results of the questionnaire on PFA implementation across the participating centers. **A:** Percentage of first-time AF ablation procedure performed using PFA. **B:** Increase in the number of first-time AF ablation procedures in relation to use of PFA. AF = atrial fibrillation; PFA = pulsed field ablation.

The results of the questionnaires showed that once this technology was adopted by the center, it quickly became the preferred technique in 57% of the centers. In addition, the short duration of the procedure enabled to increase the number of AF ablation procedures per working day in most centers (91%) (Figure 4).

## Discussion

To our knowledge, this is the first nationwide, exhaustive, and prospective registry on AF ablation procedures using the pentaspline PFA catheter. Unlike previously published registries where acute ablation success and complications were self-declared at a center level,^5^^,^^6^ our registry prospectively collected data at a patient level, ensuring high data quality. The robust methodology of the FRANCE-PFA registry has enabled us to confirm that real-life AF ablation using the pentaspline PFA catheter was acutely efficient, with short procedure times and a favorable safety profile.

### Ablation modalities

Our data provide valuable insights into current clinical practices involving PFA across a variety of centers, including nonacademic and community hospitals. In contrast to the MANIFEST PF^5^^,^^13^ or EUPORIA registries,^6^ which primarily reflected the practices of expert centers, our study captures a broader, more representative sample of real-world PFA application. Of interest, 70% of the patients received more than the initially recommended number of PFA applications per vein. Although this practice is not yet supported by robust literature, it reflects clinical impressions from leading centers that have reported recurrences with PV reconnections after the standard protocol.

The ease of use and favorable safety profile of PFA have encouraged clinicians to extend its utilization beyond PVI in persistent AF (58%) and even in patients with paroxysmal AF (17.3% of the cases). The rate of extra-PV ablation sets in the overall population (35.3%) is higher than that in the MANIFEST-PF cohort, where 22% of patients underwent additional lesion sets.^5^^,^^13^ The need for a well-defined framework to guide the use of PFA beyond PVI is evident. In a retrospective analysis of the MANIFEST-PF registry, LA posterior wall ablation beyond PVI did not improve freedom from atrial arrhythmia in patients with persistent AF.^10^

### Safety

Safety events captured in this registry were very rare, with an overall freedom from major adverse event rate of 99%. Despite the fact that FRANCE-PFA registry included all first AF ablation using the pentaspline PFA catheter since its introduction in France, rates of tamponade remained very low (0.4%). Early reports of higher tamponade rates with this device may be attributed to the initial use of a straight guidewire. Unlike the MANIFEST-PF cohort,^5^ where tamponade events were predominantly observed early in each center’s experience, our registry showed that tamponades and other major complications were evenly distributed throughout the study period (Figure 3). This suggested that these complications were more related to the inherent risks of catheter ablation in the LA rather than being specific to the pentaspline device itself.^5^

PN injury with clinical manifestation was not observed in our registry. Even though PFA is considered as a myocardial-specific energy,^4^ alterations of PN function have been reported previously. Our data were in line with the MANIFEST-17K registry,^14^ with similar incidence of PN stunning (0.04% vs 0.06%). This transient complication was certainly underdiagnosed because it was not specifically searched in all patients. A recent animal study from Howard and colleagues^15^ showed that PN stunning was frequent and dose dependent when PFA was delivered in proximity to the PN in the SVC. Notwithstanding, the authors found no evidence of chronic PN injury. Altogether, those data support a reasonable safety profile of PFA delivered in the vicinity of the PN with the need for an ongoing vigilance.

Clinical pulmonary vein stenosis remained undescribed in this registry. This was in keeping with previous reports that used magnetic resonance imaging before ablation and 3 months after ablation to assess PV narrowing, finding no clinical stenosis in any patients^16^ but greater incidence of mild or moderate narrowing in patients assigned to thermal ablation than those assigned to PFA.^17^

This study reported 7 stroke or TIA events (0.13%). This was slightly lower than the rate reported by the large randomized controlled trial ADVENT (0.3%),^16^ single-arm clinical trial PULSED-AF^18^ (0.3%), and other registries (EU-PORIA^6^ [0.57%] and MANIFEST [0.24%]).^5^ One could hypothesize that PFA might be less prothrombotic than thermal energy applied to the LA due to lesser endothelial damage. However, no pathophysiological studies have specifically investigated this point to date.

### Unexpected complications

The growing use of PFA for additional lesion sets has led to the emergence of new and unexpected complications, as documented in recent literature.^8^^,^^11^^,^^19^^,^^20^ In our cohort, only 4 cases of coronary spasms were observed from 659 patients (0.6%) who underwent mitral or CTI ablation with PFA, which is lower than the 2 spasms from 45 patients (4.4%) reported in the seminal paper evaluating the feasibility and safety of MI ablation with PFA.^8^ As recent reports suggested that use of preemptive nitroglycerin can significantly reduce the risk of vasospasm,^21^ it has now become standard practice before applying PFA along the cavotricuspid or MI, which could explain our results.

There were no documented cases of renal insufficiency related to hemolysis. It is important to note that all cases of acute renal insufficiency due to hemolysis reported by French centers have occurred secondary to redo procedures, which were not included in the current registry.^11^ Delivery of >70 PFA applications in a single procedure was found to be associated with a substantial risk of hemolysis.^11^^,^^22^ This number of applications was reached in 14% of our patient cohort. If >70 PFA was delivered, pre- and postprocedure fluid infusion could have been performed for recent cases as it has been demonstrated to strongly mitigate the risk of hemoglobinuria and renal insufficiency.^23^^,^^24^

### Efficiency

This registry captured an acute PVI success rate of 99.8%, with complications causing incomplete isolation in 8 patients, and challenging anatomy preventing right inferior PV isolation in 4 patients. This success rate was consistent with previous studies, which reported success rates close to 100%.^16^^,^^25^ This extensive patient-level data set suggested that the high success rate will likely generalize well to all centers, populations, and geographies. Notably, the only technical failures in PV isolation were with right inferior PV. Careful trans-septal puncture, avoiding the infero-posterior quadrant of the septum, may facilitate access to the right inferior pulmonary vein in patients with challenging anatomy.

Procedure times were comparable to previous registries and suggested that real-world use resulted in notably faster times than those reported in large randomized controlled trial. For instance, the ADVENT and SPHERE-AF studies reported PFA arm procedure times of 106 and 101 minutes, respectively.^16^^,^^26^ Additionally, it is important to note that the present registry includes large percentage of patients who received PFA applications beyond PVI. These procedure times were already short from the start of experience, with an average <1 hour for PVI-only procedures during the first 50 procedures per center.

### Limitations

This study is a registry-based analysis, which inherently limited the ability to enforce specific procedural protocols beyond standard care practices. As this study was limited to standard of care, the lack of specific assessments for detecting subclinical hemolysis, nonclinical PN palsy, and other silent complications may have led to an underestimation of some nonclinical adverse events. Nevertheless, this study represented the largest patient-level prospective all-comer registry of its kind and thus may generalize better to real-world conditions than controlled trials with limited lesion set and excluded patients. Finally, this study was limited to French patients, and findings may not fully generalize to other health care systems or populations with different demographics.

## Conclusion

In this prospective and exhaustive registry, AF ablation using the pentaspline PFA catheter seemed to be safe and acutely efficient despite significant variability in the number of patients treated across the centers. FRANCE-PFA is the first nationwide registry providing real-life data on this new ablation modality, which is becoming increasingly important in the field of AF catheter ablation.
